# Zoning inside the renal fascia: The anatomical relationship between the urinary system and perirenal fat

**DOI:** 10.1111/iju.14248

**Published:** 2020-04-20

**Authors:** Atsuhiko Ochi, Satoru Muro, Takuya Adachi, Keiichi Akita

**Affiliations:** ^1^ Department of Clinical Anatomy Tokyo Medical and Dental University Tokyo Japan; ^2^ Department of Urology Kameda Medical Center Chiba Japan; ^3^ Department of Radiology Kameda Medical Center Chiba Japan

**Keywords:** perinephric vein, perirenal fat, renal fascia, retroperitoneal anatomy, retroperitoneoscopy

## Abstract

**Objectives:**

To examine the anatomical relationship between the urinary system and perirenal fat, and to clarify the zoning inside the renal fascia.

**Methods:**

Using computed tomography images from 50 men, we examined perinephric veins to reveal vessel communication in perirenal fat. Nine cadavers were dissected to investigate connective tissue continuity and vessel communication inside the renal fascia. Eight retroperitoneal specimens were macroscopically observed: four from the anterior and four from the posterior aspects. One specimen was used to obtain retroperitoneal transverse sections to study macroscopic anatomy and histology.

**Results:**

Perinephric veins were classified into four types (superior, middle, inferior and lateral) using computed tomography. Most of the inferior perinephric veins were connected to the ipsilateral gonadal vein. In the cadaveric study, the superior and middle perinephric veins communicated with veins deriving from the ipsilateral adrenal gland. A fibrous connective tissue gap between perirenal fat and renal hilar fat was observed in posterior aspect dissection. From the gap, we could dissect the urinary system from perirenal fat en bloc along with a thin fibrous connective tissue layer. Communicating vessels between perirenal fat and the urinary system were rare.

**Conclusions:**

Our results suggest that perirenal fat belongs to the connective tissue of the gonad and the adrenal gland. The urinary system is separate from perirenal fat, and is located on the dorsal side inside the renal fascia. This concept of zoning inside the renal fascia is valuable particularly in retroperitoneoscopic donor nephrectomy.

**Abbreviations & Acronyms**
3‐D: three‐dimensional
CT: computed tomography
IPV: inferior perinephric vein
IVC: inferior vena cava
LPV: lateral perinephric vein
MPV: middle perinephric vein
SPV: superior perinephric vein

## Introduction

Regarding kidney transplantation, some parts of connective tissue around the donor urinary system require preservation, whereas other components do not. To avoid ureteral complications as a result of ureteral ischemia and necrosis, renal hilar, and periureteral fat containing ureteral feeding vessels should be preserved.^1^, ^2^, ^3^, ^4^ However, perirenal fat surrounding the renal capsule is removed before transplantation, as it is considered unnecessary. Thus, understanding anatomical structures inside the renal fascia is crucial in donor nephrectomy.

Anatomical reports have described the renal fascia as a membranous structure consisting of fibrous connective tissue covering the kidney and perirenal fat, as well as the ipsilateral ureter, the adrenal gland, and gonadal vessels from the anterior and posterior aspects in the retroperitoneum.^5^, ^6^, ^7^, ^8^ Since the advent of CT, the renal fascia has been recognized as a multilayered membranous structure with a potential inner space, referred to as the interfascial plane,^9^, ^10^, ^11^ rather than as a single membrane. Furthermore, bridging septa composed of a thin fibrous connective tissue septum in perirenal fat and communicating with the interfascial plane of the renal fascia have been described.^12^, ^13^ However, these interfascial planes and bridging septa were reportedly radiological concepts, and anatomical structures inside the renal fascia have not been thoroughly studied.

**Fig. 1:**
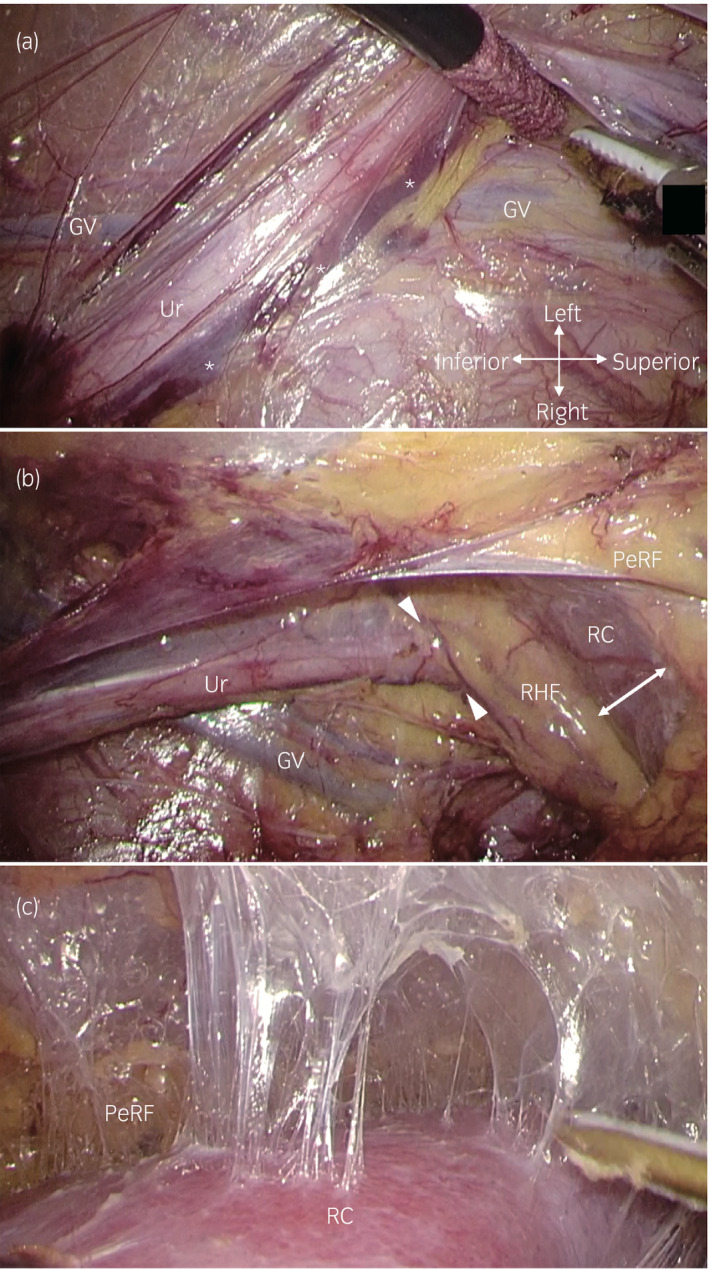
Intraoperative retroperitoneoscopic view of the left donor nephrectomy in a 61‐year‐old man. (a) The Ur, periureteral fat and ureteral feeding vessels (asterisks) were covered by a thin fibrous connective tissue sheath. This ureteral sheath was observed to exist separately behind PeRF, including the GV. (b) The ureteral sheath connected to the thin fibrous connective tissue around RHF (arrowheads). The RC was initially exposed to a fibrous connective tissue gap between PeRF and RHF (two‐way arrow). (c) PeRF was separated from the RC along with the thin fibrous connective tissue layer. Communicating vessels between PeRF and the RC were rare. GV, gonadal vein; PeRF, perirenal fat; RC, renal capsule; RHF, renal hilar fat; Ur, ureter.

Embryological knowledge and retroperitoneoscopic surgery observations might provide hints regarding the understanding of anatomical structures inside the renal fascia. In embryology, the kidney and the ureter developing from the metanephros and the mesonephric duct ascend from the sacral region.^14^ In retroperitoneoscopic surgery, the urinary system is observed to exist posterior to perirenal fat, including gonadal vessels inside the renal fascia (Fig. 1). From the perspective of embryology and retroperitoneoscopic surgery observations, we hypothesized that anatomical zoning exists inside the renal fascia: the urinary system is separated from perirenal fat and is located on the dorsal side. Laparoscopic and retroperitoneoscopic surgeries have been widely carried out in donor nephrectomy for living kidney transplantation.^15^, ^16^, ^17^ If the zoning inside the renal fascia is revealed, this anatomical knowledge would be valuable for the selection of appropriate and efficient dissection layers around the urinary system in donor nephrectomy.

This study aimed to examine the anatomical relationship between the urinary system and perirenal fat by investigating vessel communication and connective tissue continuity, and to clarify the zoning inside the renal fascia.

## Methods

### CT study

We retrospectively evaluated the enhanced abdominal CT images from 50 men (mean age 68.5 years; range 44–88 years). Between August 2014 and April 2019, CT was carried out at Kameda Medical Center for the pretreatment evaluation of prostate cancer (*n* = 36), as well as for either pretreatment evaluation before transurethral resection of bladder cancer or follow‐up examination subsequent to transurethral resection of bladder cancer (*n* = 14). We excluded any congenital abnormalities of the upper urinary system, including an ectopic or horseshoe kidney, upper urinary neoplasm or abdominal lymph node metastasis, prior history of retroperitoneal surgery and other retroperitoneal abnormalities that could potentially affect the retroperitoneal structures. In addition, we excluded women, because the ovaries and urinary system are close in proximity, and evaluating vessel communication between the perinephric vein and the gonadal vein could prove challenging.

We injected 1.8 mL/kg of an intravenous non‐ionic contrast material (iohexol, Omnipaque 300; Daiichi Pharmaceutical, Tokyo, Japan) as a bolus in all patients. CT examinations were carried out using 64‐detector CT scanners (Aquilion; Toshiba, Tokyo, Japan). All scanning procedures were undertaken at 100 s after the completion of contrast injection. Excretory phase scanning was also carried out at 6 min after contrast injection in patients with bladder cancer. CT parameters were as follows: tube voltage, 120 kv; tube current, 102–200 mA/s; collimation, 32 mm; pitch, 0.828; field of view, 320–435 mm; and matrix, 512 × 512. Axial images were reconstructed at 2‐mm slice thickness. Perirenal veins were evaluated using a picture archiving and communication system (SYNAPSE Enterprise‐PACS; Fujifilm, Tokyo, Japan). 3‐D reconstructed images of the excretory phase were obtained from two patients with bladder cancer (two men aged 45 and 60 years) using a 3‐D workstation (Ziostation 2; Ziosoft, Tokyo, Japan). All CT examinations were reviewed by TA, a radiologist with 5 years of experience, and AO, a urological surgeon with 14 years of experience.

### Cadaveric study

The cadavers used in the present study were donated to the Department of Anatomy, Tokyo Medical and Dental University, Japan. The present study was carried out in accordance with the Japanese law, “Act on Body Donation for Medical and Dental Education.” All donors voluntarily declared before their deaths that their remains were to be donated for educational purposes. This voluntary donor system for cadavers is applied throughout Japan, and the present study entirely complies with the current laws of Japan. The cadavers were fixed by arterial perfusion with 8% formalin, and preserved in 30% alcohol to prevent fungal growth and maintain tissue softness.

Nine cadavers (four men and five women; mean age 77.7 years at death; range 60–94 years) with no congenital and/or acquired abnormalities in their retroperitoneal structures were used for dissection. Nine retroperitoneal specimens, including adrenal glands, kidneys, ureters, the aorta, the IVC and retroperitoneal connective tissues, were obtained en bloc. Four retroperitoneal specimens (two men and two women; mean age 73.3 years at death; range 60–92 years) were dissected from the posterior, and another four retroperitoneal specimens (one man and three women; mean age 83.3 years at death; range 73–94 years) were dissected from the anterior aspect. From the posterior aspect, the skin, muscles and vertebral bodies were removed, and the posterior renal fascia was visualized. From the anterior aspect, the abdominal organs and peritoneum were removed, and the anterior renal fascia was visualized.

Another retroperitoneal specimen (one man aged 73 years at death) was used for sectional analysis and histology. The specimen, including the upper and lower poles of the perirenal fat, was frozen at −80ºC and cut into 10‐mm thick transverse sections, from which small blocks were obtained for histology. The blocks were fixed in 10% formalin, dehydrated and embedded in paraffin. After cutting into 5‐μm thick sections, the histological sections were stained with Elastica van Gieson stain and Masson’s trichrome stain.

### Ethical approval

The study was approved by the Board of Ethics of Kameda Medical Center (19‐032‐190725) and Tokyo Medical and Dental University (M2019‐075).

## Results

### Vessel communication in perirenal fat

**Fig. 2:**
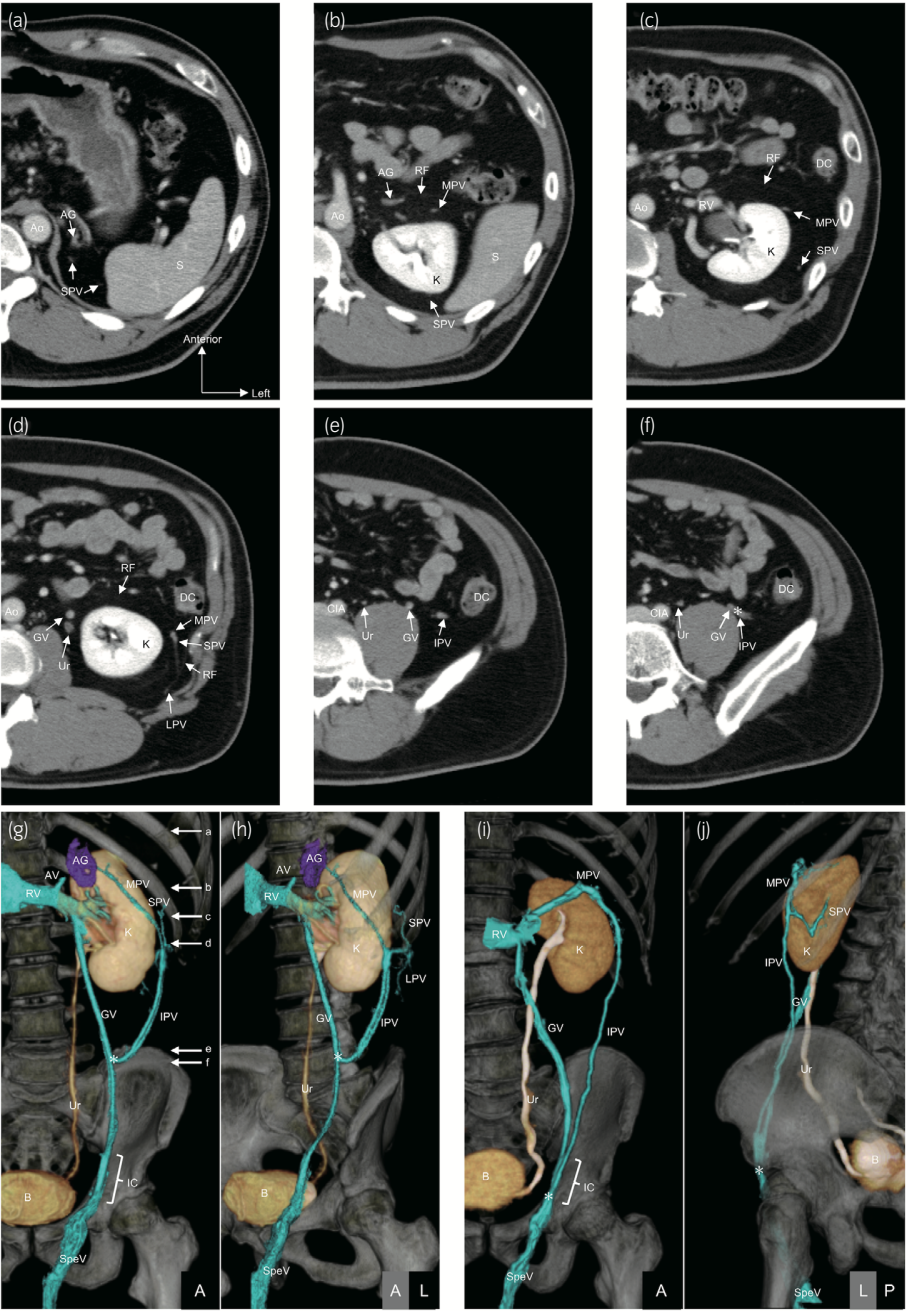
CT images of perinephric veins. (a–f) CT images (axial view) of a 45‐year‐old man. The IPV connected to the GV (asterisk). (g,h) 3‐D reconstructed CT images of a 45‐year‐old man (the same patient in a–f; arrows a–f in g correspond to the section in a–f). The MPV connected to the left emissary vein of the AG and the IPV to the GV at the lower pole of perirenal fat (asterisk). The superior and LPVs were poorly reconstructed. (i,j) 3‐D reconstructed CT images of a 60‐year‐old man. The MPV connected to the left RV and the IPV to the GV in the IC (asterisk). The superior and LPVs were poorly reconstructed. AG, adrenal gland; Ao, aorta; AV, adrenal vein; B, bladder; CIA, common iliac artery; DC, descending colon; GV, gonadal vein; IC, inguinal canal; K, kidney; RF, renal fascia; RV, renal vein; S, spleen; SpeV, spermatic vein; Ur, ureter.

Axial CT images of 50 left and right sides from 50 men were evaluated (Fig. 2a–f). Perinephric veins in perirenal fat were classified into four types: the SPV, which runs at the upper pole of perirenal fat (left: 49/50, 98%; right: 50/50, 100%); the MPV, which runs on the anterior side of the kidneys (left: 50/50, 100%; right: 47/50, 94%); the IPV, which runs at the lower pole of perirenal fat (left: 50/50, 100%; right: 50/50, 100%); and the LPV, which runs from the body wall muscle and pararenal fat through the renal fascia (left: 46/50, 92%; right: 45/50, 90%). Every vein connected on the lateral side of the kidneys. Those veins were reconstructed in 3‐D images; however, reconstructions of the SPV and the LPV were poor, because these veins were smaller than the MPV and the IPV (Fig. 2g–j).

**Fig. 3:**
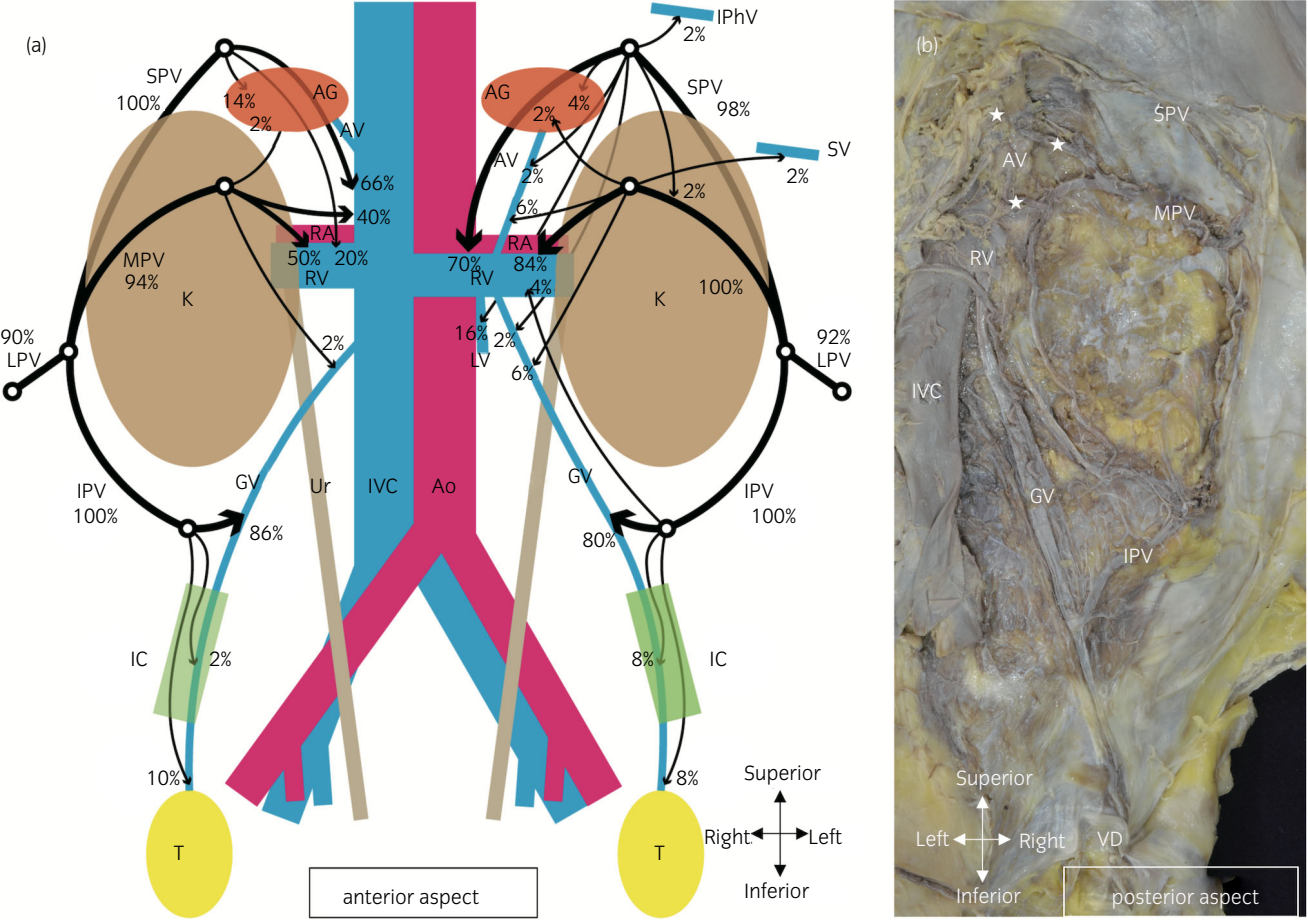
Schema and macroscopic anatomy of perinephric veins. (a) Anterior aspect. Schema of perinephric veins distribution and frequency. Numbers in this figure represent the frequencies of perinephric veins shown in Table 1. Line thickness refers not to the actual size, but to the frequency of veins. (b) The retroperitoneum of a male cadaver (60 years‐of‐age at death). The right urinary system and perirenal fat were removed to show perinephric veins. Several fine branches from perinephric veins were observed. Arteries ran along perinephric veins and branches. The SPV and the MPV had several communicating veins, with the veins deriving from the AG (stars). In this image, the LPV had already been removed with perirenal fat. AG, adrenal gland; Ao, aorta; AV, adrenal vein; GV, gonadal vein; IC, inguinal canal; IPhV, inferior phrenic vein; K, kidney; LV, lumbar vein; RA, renal artery; RV, renal vein; SV, splenic vein; T, testis; Ur, ureter; VD, vas deferens.

The frequencies and destinations of perinephric veins, as seen on CT, are summarized in Figure 3a and Table 1. The connection from the perinephric vein to another vein was evaluated with the largest vein. The SPV mainly connected to the renal vein or lumbar vein on the left side, and to the renal vein or the IVC on the right side (left: 43/50, 86%; right: 43/50, 86%). The MPV mainly connected to the renal vein on the left side, and to the renal vein or the IVC on the right side (left: 42/50, 84%; right: 45/50, 90%). Most of the IPV connected to the ipsilateral gonadal vein (left: 48/50, 96%; right: 49/50, 98%). In some cases, the junction was distal to the internal inguinal ring (left: 8/50, 16%; right: 6/50, 12%).

**Table 1:** Destinations and frequencies of perinephric veins by CT in 50 men

Branch	Left		Right	
	Destination	Frequency, *n* (%)	Destination	Frequency, *n* (%)
SPV		49 (98)		50 (100)
	Renal vein	35 (70)	IVC	33 (66)
	Lumbar vein	8 (16)	Renal vein	10 (20)
	Emissary vein of adrenal gland	2 (4)	Emissary vein of adrenal gland	7 (14)
	Adrenal vein	1 (2)		
	Gonadal vein	1 (2)		
	Inferior phrenic vein	1 (2)		
	MPV	1 (2)		
MPV		50 (100)		47 (94)
	Renal vein	42 (84)	Renal vein	25 (50)
	Adrenal vein	3 (6)	IVC	20 (40)
	Gonadal vein	3 (6)	Gonadal vein	1 (2)
	Emissary vein of adrenal gland	1 (2)	Emissary vein of adrenal gland	1 (2)
	Splenic vein	1 (2)		
IPV		50 (100)		50 (100)
	Gonadal vein (region 1)	40 (80)	Gonadal vein (region 1)	43 (86)
	Gonadal vein (region 2)	4 (8)	Gonadal vein (region 2)	1 (2)
	Gonadal vein (region 3)	4 (8)	Gonadal vein (region 3)	5 (10)
	Renal vein	2 (4)	Unknown	1 (2)
LPV		46 (92)		45 (90)

Region 1, between the lower pole of the kidney and internal inguinal ring; region 2, in the inguinal canal; region 3, between the external inguinal ring and testis; lumbar vein (1st, 2nd or 3rd).

We also evaluated the frequencies and destinations of branches from the SPV, the MPV or the IPV (Table 2). Some branches connected to the mesentery (left: 6/50, 12%; right: 15/50, 30%). Few branches directly connected to the renal capsule (left: 2/50, 4%; right: 3/50, 6%), and none to the ureter (left: 0/50, 0%; right: 0/50, 0%).

**Table 2:** Destinations and frequencies of branches from perinephric veins by CT in 50 men

Destination	Left, *n* (%)	Right, *n* (%)
Renal capsule		
Yes	2 (4)	3 (6)
No	48 (96)	47 (94)
Ureter		
Yes	0 (0)	0 (0)
No	50 (100)	50 (100)
Mesentery		
Yes	6 (12)	15 (30)
No	44 (88)	35 (70)

In macroscopic anatomy, the course of perinephric veins was evaluated at the end of dissection. CT image findings were also confirmed in macroscopic anatomy. The findings for the vessel course and communication did not differ between anterior and posterior aspect dissection. The vessels in the perirenal fat mainly ran in the anterior aspect; therefore, the overall vessel course remained more clearly in the posterior aspect (Fig. 3b). Numerous fine branches of the perinephric vein were observed; the arteries were next to the veins. In all cases, numerous fine branches from the SPV and the MPV communicated with veins deriving from the ipsilateral adrenal gland (stars in Fig. 3b).

### Connective tissue continuity and vessel communication of the urinary system and perirenal fat

**Fig. 4:**
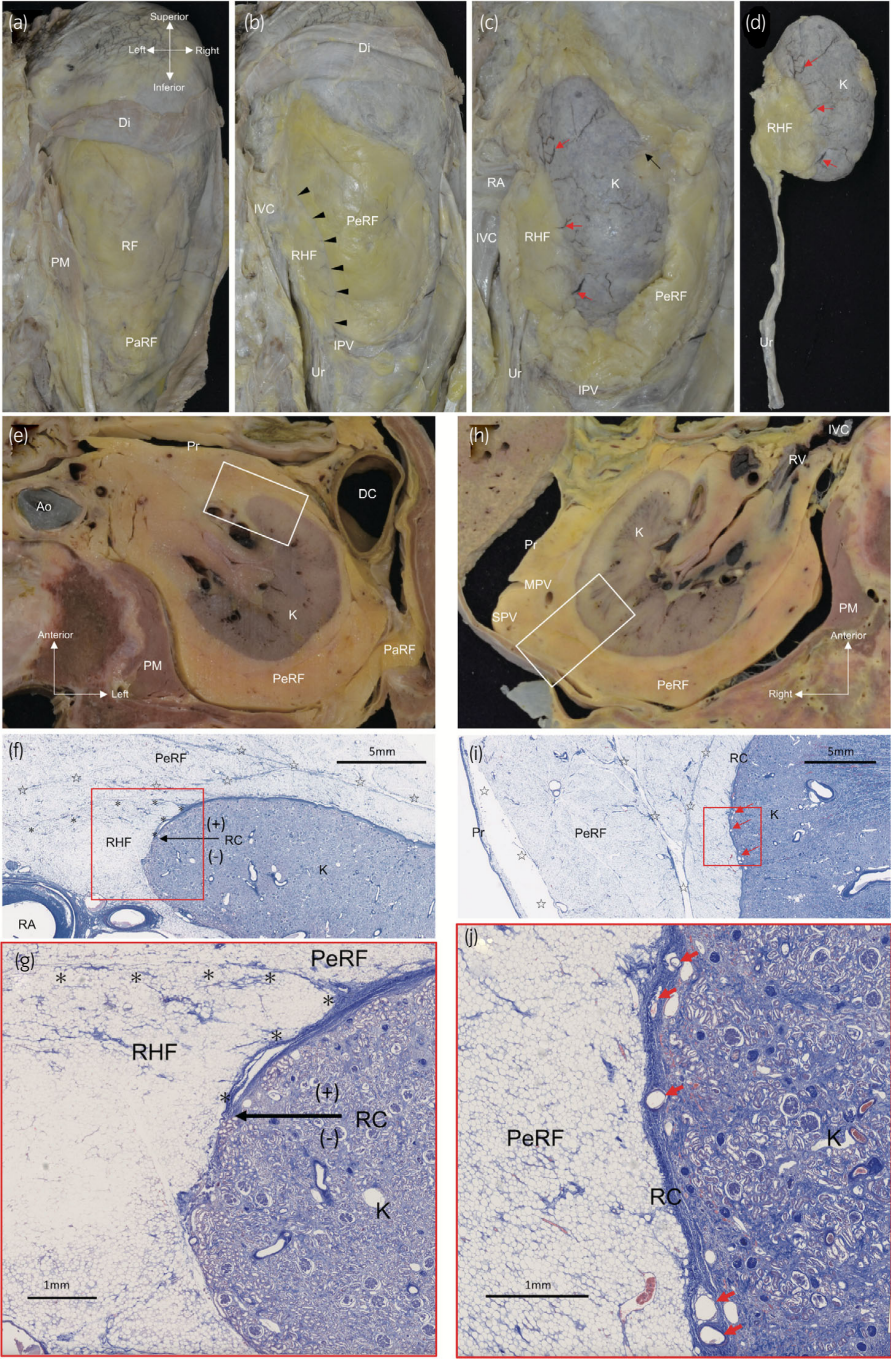
Macroscopic anatomy and histology of the retroperitoneum. (a–d) The retroperitoneum of a female cadaver (78 years‐of‐age at death) observed from the posterior aspect. (a) Macroscopic anatomy of the right retroperitoneum from the posterior aspect after removal of the skin, muscles and vertebral bodies. (b) PaRF and the posterior RF were removed. A fibrous connective tissue gap was observed between PeRF and RHF (arrowheads). (c) From the gap, the posterior aspect of PeRF was removed, along with the thin fibrous connective tissue layer around the RC. The renal capsular veins ran on the surface of the kidney and continued to the veins in RHF (red arrows). Communicating vessels from PeRF to the RC were few (black arrow). (d) The right urinary system was removed en bloc with RHF. The Ur, periureteral fat and ureteral feeding vessels were covered by a thin fibrous connective tissue sheath. Ureteral feeding vessels communicated with the vessels in RHF. The ureteral sheath continued to the thin fibrous connective tissue around RHF. The renal capsular veins ran on the surface of the RC and continued to veins in RHF (red arrows). (e–j) The retroperitoneum of a male cadaver (73 years‐of‐age at death) observed in transverse section. (e) A transverse section of the left retroperitoneum. (f) Histology of the white square section in Figure 3e (Masson’s trichrome stain). (g) High magnification of the red square section in Figure 3f (Masson’s trichrome stain). The RC could be confirmed between PeRF and the renal cortex, but not between RHF and the renal cortex (the transition point is indicated by a black arrow). Stars (☆), bridging septa; asterisks (＊), border of the RHF. (h) A transverse section of the right retroperitoneum. (i) Histology of the white square section in Figure 3h (Masson’s trichrome stain). (j) High magnification of the red square section in Figure 3i (Masson’s trichrome stain). The renal capsular veins ran in the internal space of the RC (red arrows). Communicating vessels between PeRF and the RC were not identified. Stars (☆), bridging septa. Ao, aorta; DC, descending colon; Di, diaphragm; GV, gonadal vein; K, kidney; PaRF, pararenal fat; PeRF, perirenal fat; PM, psoas muscle; Pr, peritoneum; RA, renal artery; RC, renal capsule; RHF, renal hilar fat; RV, renal vein; Ur, ureter.

Regarding posterior aspect dissection (Fig. 4a), we removed the pararenal fat and the posterior renal fascia. A fibrous connective tissue gap was observed between perirenal and renal hilar fat (arrowheads in Fig. 4b). From the gap, the posterior aspect of perirenal fat was removed along with a thin fibrous connective tissue layer around the renal capsule. Few communicating vessels between perirenal fat and the renal capsule were observed in the thin fibrous connective tissue. The renal capsular veins ran on the surface of the kidney and continued to the veins in renal hilar fat (red arrows in Fig. 4c). After cutting of the renal artery and vein, the anterior aspect of perirenal fat was also removed from both renal hilar fat and the renal capsule, along with the thin fibrous connective tissue layer. Communicating vessels were also rare in the thin fibrous connective tissue. The urinary system was removed en bloc with renal hilar fat (Fig. 4d). The ureter, periureteral fat and ureteral feeding vessels were covered by a thin fibrous connective tissue sheath. The ureteral feeding vessels communicated with the vessels in renal hilar fat. The ureteral sheath continued to the thin fibrous connective tissue around renal hilar fat. In macroscopic anatomy and histology of the retroperitoneal transverse sections, renal hilar fat connected to the renal cortex, where the renal capsule was not observed (Fig. 4e–g). The renal capsular veins ran in the internal space of the renal capsule (Fig. 4h–j). Communicating vessels between perirenal fat and the renal capsule were not identified.

**Fig. 5:**
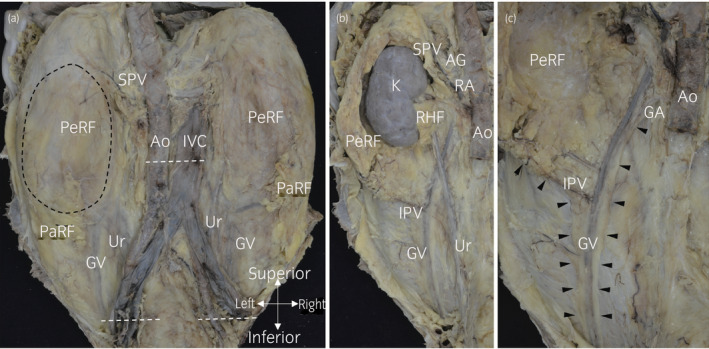
The retroperitoneum of a male cadaver (63 years‐of‐age at death) observed from the posterior aspect. (a) The macroscopic anatomy of the retroperitoneum from the posterior aspect after removal of the skin, back muscles and vertebral bodies. (b) The left retroperitoneal posterior aspect. The Ao, the IVC, and external and internal vessels were cut along the white dashed lines shown in (a) and removed. The posterior side of PeRF was removed along the black dashed circle shown in (a). The Ur, periureteral fat and ureteral feeding vessels were covered by a thin fibrous connective tissue sheath. The GV and the IPV connected at the lower pole of PeRF. The ureteral sheath ran separately behind PeRF. (c) After removing the left urinary system, PeRF converged toward the caudal side along the gonadal vessels (arrow heads). AG, adrenal gland; Ao, aorta; GA, gonadal artery; GV, gonadal vein; K, kidney; PaRF, pararenal fat; PeRF, perirenal fat; RA, renal artery; RHF, renal hilar fat; RV, renal vein; Ur, ureter.

Furthermore, in posterior aspect dissection (Fig. 5a), the ureter, periureteral fat and ureteral feeding vessels, surrounded by a ureteral sheath, ran separately behind perirenal fat (Fig. 5b). The gonadal vein and the IPV ran in perirenal fat and connected at the lower pole of perirenal fat. After removing the urinary system, perirenal fat converged along the gonadal vessels from the confluence of the gonadal vein and the IPV toward the caudal side (Fig. 5c).

We also attempted to dissect the anterior aspect. The fibrous connective tissue gap between perirenal fat and renal hilar fat was not observed from the anterior aspect. On the cranial side of the crossing point of the gonadal vein and the ureter, a ureteral sheath was not exposed initially and not identified, because renal hilar fat and periureteral fat were covered anteriorly with perirenal fat.

## Discussion

The present study examined the anatomical relationship between the urinary system and perirenal fat to clarify the zoning inside the renal fascia. We investigated vessel communication and connective tissue continuity inside the renal fascia by CT and cadaveric studies. The results showed that vessel communications were observed between perinephric veins and the vessels deriving from the adrenal gland, perinephric veins and the gonadal vein, the renal capsular veins and veins in renal hilar fat, the ureteral feeding vessels, and vessels in the renal hilar fat. However, vessel communications were rare between perinephric veins and the urinary system. Regarding connective tissue continuity, the renal capsule, periureteral and renal hilar fat were surrounded by a continuous thin fibrous connective tissue. By contrast, the urinary system was separated from perirenal fat by the thin fibrous connective tissue. A sheath structure around the ureter, and a gap between perirenal and renal hilar fat were initially observed during posterior aspect dissection.

In 1883, Zuckerkandl discovered the posterior renal fascia, and in 1895, Gerota reported the anterior renal fascia as a membranous structure consisting of fibrous connective tissue that covers the kidney and perirenal fat.^18^ Hence, the posterior renal fascia is called Zuckerkandl’s fascia, whereas the anterior renal fascia is referred to as Gerota’s fascia, which is also used as a general term that pertains to both the anterior and posterior renal fascia. A consensus regarding renal fascia continuity between the left and right sides, toward the pelvic side, and around the aorta and the IVC has yet to be reached. Nevertheless, the renal fascia is recognized as an important anatomical structure when considering the compartment in the retroperitoneum.^5^ According to recent surgical and anatomical textbooks, the anterior and posterior renal fascia surrounds not only the kidney and perirenal fat, but also the ipsilateral ureter, the adrenal gland and gonadal vessels.^6^, ^7^, ^8^ Although the anatomical structures inside the renal fascia have not been thoroughly examined, radiological studies have divided the retroperitoneum into three spaces – namely, anterior pararenal, perirenal and posterior pararenal spaces.^19^ Since the advent of CT, the concept of “interfascial plane” has been reported, with the renal fascia having been recognized as a multilayered membranous structure with a potential inner space rather than as a single membrane.^9^, ^10^ Furthermore, the potential spaces within the anterior and posterior renal fascia have been described to continue through fibrous connective tissue pathways in perirenal fat called “bridging septa.”^12^, ^13^ In recent years, a theory proposing that the potential spaces within the renal fascia continue to the spaces around pararenal fat has been suggested.^11^ The bridging septa indicate the zoning structure inside the renal fascia. However, these ideas were designed from retroperitoneal inflammation and fluid movement, and were not anatomical revelations of the structure inside the renal fascia. This study examined the anatomical relationship between the urinary system and perirenal fat to anatomically clarify the zoning inside the renal fascia. The retroperitoneal anatomy has been previously investigated based on the membranous structure by anterior dissection. However, we focused on and examined both the vessel communication and the connective tissue continuity. Furthermore, we obtained hints from retroperitoneoscopic surgical findings and also dissected from the posterior aspect.

**Fig. 6:**
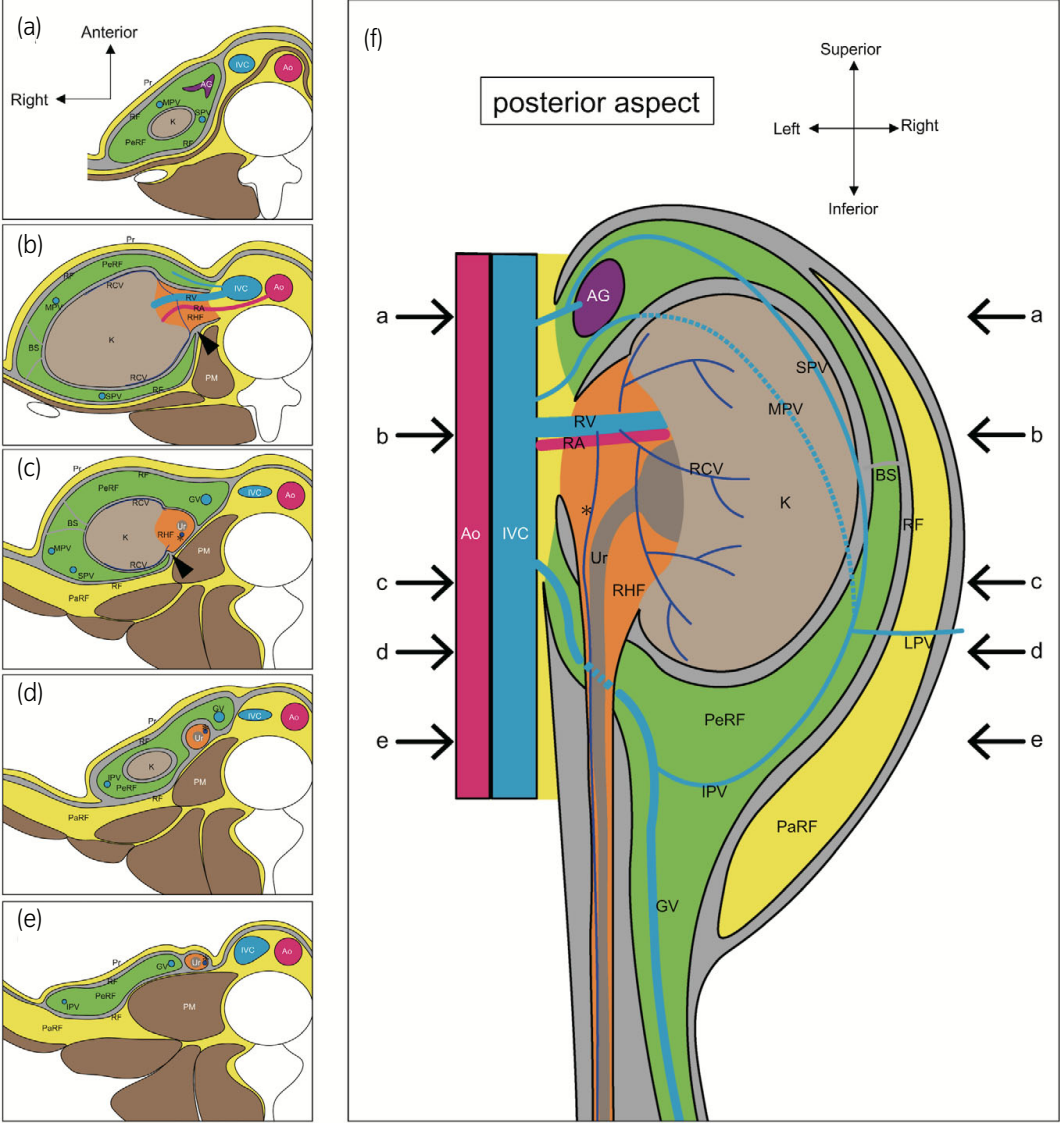
A new anatomical concept of zoning inside the RF. These figures are drawn based on the concept of “interfascial plane.”^9^, ^10^, ^11^ The gray area represents a potential space within a multilayered membranous structure. (a–e) Transverse sections of parts indicated by arrows a–e in (f). The urinary system, RHF and periureteral fat (orange area) were separated from PeRF (green area) by a thin fibrous connective tissue (gray area). A gap between PeRF and RHF is indicated by an arrowhead in (b,c). The Ur, periureteral fat and ureteral feeding vessels were covered by a thin fibrous connective tissue sheath. The ureteral feeding vessel is indicated by an asterisk in (c–f). AG, adrenal gland; Ao, aorta; BS, bridging septum; GV, gonadal vein; K, kidney; PaRF, pararenal fat; PeRF, perirenal fat; PM, psoas muscle; Pr, peritoneum; RA, renal artery; RCV, renal capsule vein; RF, renal fascia; RHF, renal hilar fat; RV, renal vein; Ur, ureter.

Connective tissue continuity and vessel communication indicate the affinity of tissues. By contrast, connective tissue boundary and rarity of vessel communication indicate the separation between tissues. The present results suggest that perirenal fat belongs to the connective tissue of the gonad and the adrenal gland. Furthermore, the renal capsule, renal hilar fat and periureteral fat were surrounded by a continuous thin fibrous connective tissue and affiliated to the urinary system. The urinary system was located on the dorsal side of perirenal fat. Therefore, we propose a new anatomical concept of zoning inside the renal fascia (Fig. 6). This concept is useful for understanding the structures around the urinary system in surgeries, especially for retroperitoneoscopic donor nephrectomy in living donor kidney transplantation, to recognize efficient dissection layers and preserve blood supply to the ureter. It also provides new suggestions for the development of the urinary system, which is known to ascend from the sacral region during the embryological period. Our concept suggests that the urinary system ascends at the posterior region inside the renal fascia.

However, the present study had some limitations. First, it was not validated in fetuses, as only elderly cadavers were observed as a result of fetal cadaver rarity. Second, only men were evaluated in the CT study, as it was difficult to investigate the relationship between perinephric veins and the gonadal vein in women due to the ovaries and the urinary system being in close proximity.

In conclusion, connective tissue continuity and vessel communication showed an anatomical relationship between the urinary system and perirenal fat. The present results suggest that anatomical zoning exists inside the renal fascia: the urinary system is separate from perirenal fat and is located on the dorsal side.

## Conflict of interest

None declared.
